# A stepped-wedge randomized trial investigating the effect of the Leadership and Organizational Change for Implementation (LOCI) intervention on implementation and transformational leadership, and implementation climate

**DOI:** 10.1186/s12913-022-07539-9

**Published:** 2022-03-04

**Authors:** Ane-Marthe Solheim Skar, Nora Braathu, Nadina Peters, Harald Bækkelund, Mathilde Endsjø, Aida Babaii, Randi Hovden Borge, Tore Wentzel-Larsen, Mark G. Ehrhart, Marisa Sklar, C. Hendricks Brown, Gregory A. Aarons, Karina M. Egeland

**Affiliations:** 1 Norwegian Centre for Violence and Traumatic Stress Studies (NKVTS), Gullhaugveien 1-3, 0484 Oslo, Norway; 2 Centre for Child and Adolescent Mental Health, Eastern and Southern Norway, Gullhaugveien 1, 0484 Oslo, Norway; 3 Department of Psychology, University of Central Florida, 4111 Pictor Lane, Orlando, FL 32816-1390 USA; 4 Department of Psychiatry, University of California, San Diego, 9500 Gilman Drive (0812), La Jolla, San Diego, CA 92093-0812 USA; 5 Child and Adolescent Services Research Center, 3665 Kearny Villa Rd., Suite 200N, San Diego, CA 92123 USA; 6 Feinberg School of Medicine, Northwestern University, 750 North Lake Shore Drive, Chicago, IL 60611 USA

**Keywords:** Evidence-based practice, LOCI, Mental health, Implementation climate, Implementation strategies, PTSD, Transformational leadership, Implementation leadership

## Abstract

**Background:**

This study evaluates the Leadership and Organizational Change for Implementation (LOCI) strategy and its effect on implementation leadership, transformational leadership, and implementation climate.

**Methods:**

A stepped wedge cluster randomized study design enrolling 47 first-level leaders from child- and adult-specialized mental health clinics within Norwegian health trusts across three cohorts. All therapists (*n* = 790) received training in screening of trauma exposure and posttraumatic stress, and a subgroup of therapists (*n* = 248) received training in evidence-based treatment methods for posttraumatic stress disorder (PTSD). First-level leaders and therapists completed surveys at baseline, 4, 8-, 12-, 16-, and 20-months assessing leadership and implementation climate. General linear mixed-effects models were used to investigate whether the LOCI strategy would lead to greater therapist-rated scores on implementation leadership, transformational leadership, and implementation climate.

**Results:**

After introducing the LOCI strategy, there was a significant increase in therapist-rated implementation and transformational leadership and implementation climate. The increase was sustained at all measurement time points compared to non-LOCI conditions, which demonstrated a steady decrease in scores before LOCI.

**Conclusions:**

The LOCI strategy can develop better transformational and implementation leadership skills and contribute to a more positive implementation climate, which may enhance successful EBP implementation. Thus, LOCI can help leaders create an organizational context conducive for effective EBP implementation.

**Trial registration:**

Retrospectively registered: ClinicalTrials NCT03719651, 25th of October 2018.

The trial protocol can be accessed from https://www.ncbi.nlm.nih.gov/pmc/articles/PMC6417075/.

**Supplementary Information:**

The online version contains supplementary material available at 10.1186/s12913-022-07539-9.

## Background

Leadership has consistently been highlighted as important for achieving successful evidence-based practice (EBP) implementation and sustainment [1–4]. Leader behaviors are associated with a range of positive outcomes at multiple health systems and organization levels [5], such as fostering positive staff attitudes [6], lowering staff turnover [7], improving organizational climate and therapeutic alliance [8, 9], and increasing patient satisfaction and quality of life [10, 11]. Thus, leadership development is promising for facilitating improvements in the delivery of healthcare services. Although leader development is a multi-billion-dollar industry globally, with many leader development programs available [12, 13], some lack research evidence [14], and many fail to fulfill expectations for improvements in organizational effectiveness [15]. In addition, few leader development programs have highlighted specific strategies that organizations and leaders can use to align efforts to improve implementation outcomes. However, some strategic approaches can facilitate more effective leadership development [16]. Research and evaluation are needed to support their effectiveness and strengthen leadership for EBP implementation in health care settings.

The Leadership and Organizational Change for Implementation (LOCI) strategy [17, 18] is a leader development program focused on implementing specific evidence-based practices (EBPs) in healthcare services. LOCI serves as an implementation strategy that aims to build leadership skills and create a positive strategic organizational climate to support effective and sustained implementation of EBPs (see full description of LOCI; [18, 19]). LOCI targets first-level leaders responsible for supervising individuals providing direct services, while simultaneously including executive management to facilitate an aligned implementation approach [20]. By training first-level leaders in LOCI, it is hypothesized that they will exhibit more transformational and implementation leadership. In addition, LOCI encourages the development of systems and procedures to support EBP implementation. Consequently, as employees experience their leadership’s support of implementation and the systems and procedures are aligned around implementation effectiveness, they are more likely to report a positive and supportive unit-level implementation climate [18, 20, 21].

LOCI utilizes two central leader development theories. *The Full-Range Leadership Model (FRL)* targets general leadership skills and behaviors that create a shared vision and positive work environment so that staff may feel emotionally and intellectually engaged. FRL is well researched and validated globally [22] and involves transformational and transactional leadership and non-leadership (e.g., laissez-faire). Transformational leaders perform four distinct behaviors: inspirational motivation, idealized influence, intellectual stimulation, and individualized consideration. Transformational leadership has shown to be favorably related to a variety of employee and organizational outcomes, such as employees’ job satisfaction [23], perceived job demands and turnover intentions [7], organizational climate and work engagement [23, 24], as well as the adoption, use, and success of EBP implementation [25]. Although LOCI places a heavier emphasis on improving transformational leadership relative to transactional leadership, transactional leadership is also included. Specifically, the contingent reward dimension of transactional leadership is related to a leader’s ability to manage and motivate their employees through appropriate rewards [22]. *Implementation leadership* theory hypothesizes that leaders will achieve better implementation outcomes when they are proactive, knowledgeable, supportive, and perseverant about implementing specific EBPs [21]. Furthermore, LOCI also builds on theories on *implementation climate,* defined as [26] “the extent to which employees share perceptions that the adoption and implementation of the EBP are expected, supported, and rewarded within their organization” [27, 28]. Implementation climate has been shown to mediate the effect of implementation leadership on therapists’ use of EBP [26].

LOCI has been tested in one study [17], and three ongoing randomized controlled trials [18, 29] are underway in the United States, funded by the US National Institutes of Health. Preliminary results have shown that LOCI is feasible and acceptable [17] and is related to improved staff-rated leadership and implementation climate for EBP implementation [17, 18, 30]. Although there has been increased interest in approaches to leadership in implementation research and practice [31–33], there is a need for testing the effectiveness of strategies such as LOCI on implementation and transformational leadership and implementation climate in a variety of settings. Such knowledge can facilitate successful EBP implementation and sustainment. This is the first study to examine the effect of LOCI outside of the USA, potentially strengthening its generalizability. We aim to test the effect of LOCI on the factors specifically addressed in LOCI, namely implementation and transformational leadership and implementation climate compared to the non-LOCI condition. Based on theory and empirical evidence, we hypothesize that:

- H1: Implementation leadership will improve more in the LOCI as compared to the non-LOCI condition.
- H2: Transformational leadership will improve more in the LOCI as compared to the non-LOCI condition.
- H3: Implementation climate will improve more in LOCI as compared to the non-LOCI condition.

## Methods

The current study utilizes a stepped-wedge randomized design to investigate the effect of the LOCI strategy. Please see the study protocol [19] for further details about the study.

### Participants

Participants were therapists (*n* = 790) with an average age of 43.9, 75.3% were female, and approximately half were clinical psychologists (Table 1). They completed questionnaires addressing implementation climate and general and implementation leadership among first-level leaders (*n* = 47) who received the LOCI intervention at 43 participating clinics (Table 1).

**Table 1:** Participant characteristics

	LOCI leaders (***N*** = 47)	Therapists (***N*** = 804)	Overall (***N*** = 851)
**Gender**			
Women	29 (61.7%)	606 (75.4%)	635 (74.6%)
Men	18 (38.3%)	171 (21.3%)	189 (22.2%)
**Education**			
Psychology	26 (55.3%)	371 (46.1%)	397 (46.7%)
Medicine	5 (10.6%)	151 (18.8%)	156 (18.3%)
Social worker	8 (17.0%)	60 (7.5%)	68 (8.0%)
Nurse	8 (17.0%)	55 (6.8%)	63 (7.4%)
Other	0 (0%)	89 (11.1%)	89 (10.5%)
**Age**			
Mean (SD)	49.7 (7.64)	43.8 (11.1)	44.2 (11.0)
Missing	0 (0%)	115 (14.3%)	115 (13.5%)

### Setting

This study was conducted in public outpatient specialized mental health clinics for children and adolescents, and for adults. The clinics are localized within the four (North, West, South-Eastern, Central) regional health trusts across Norway, each of which consists of local health trusts. The participating clinics were from 12 different local health trusts; 67% from the South-Eastern, 19 from the Western, 9% from the Central, and 5% from the Northern health trust. This distribution is in line with the number of inhabitants in the health trusts. In 2018, the mean number of inhabitants within the participating health trusts was 175 000 (range = 34 000-295 000) [34]. The average number of therapists within each clinic was 17 (range = 8-35).

Specialized clinics offer various types of treatment related to more serious symptoms and disorders, whereas the municipalities offer services for less severe problems. Referrals to the specialized clinics are provided by the medical doctor. The public healthcare system in Norway is heavily subsidized to make it universally available. The patients often pay a small user fee for the treatment up to a certain level (approximately 200 Euros/$220), and then receive an exemption card which provides them with free treatment for the rest of the year. Children and adolescents receive fully free mental health care [35].

### Procedures

At baseline, all therapists (*n* = 790) in the participating clinics were trained to screen and diagnose PTSD (Table 2). In addition, a sub-group of therapists (*n* = 249) received training in three of the most well-documented EBPs for PTSD [36, 37], namely Trauma-Focused Cognitive-Behavioral Therapy [38] for children, and either the Eye Movement Desensitization and Reprocessing [39] or Cognitive Therapy for PTSD [40] for adults. The CT-PTSD and EMDR training consisted of a three-day course followed by 10 h coaching group calls divided by 2 h once a month for 5 months. Specialists gave training and supervision in each of the three EBPs. The TF-CBT training included 3 days of initial training followed by weekly 1-h case coaching calls in groups for a year (approximately 40 h). Following the training, all clinics were eligible to screen patients and provide EBP for PTSD.

**Table 2:**
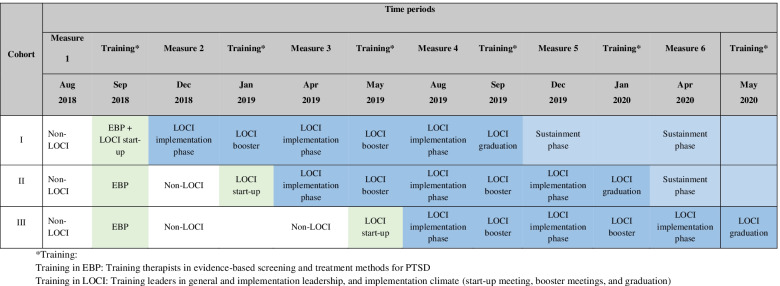
Stepped-wedge study design

Forty-eight first-level leaders from 48 different child and adolescent (*n* = 26) and adult (*n* = 22) clinics were randomized by a computer algorithm into one of three cohorts, each initiating training in LOCI at three different time points as indicated in the stepped-wedge (Table 2). The stratified randomization was made based on the following variables: number of therapists per clinic, co-localization of more than one clinic, number of therapists to receive training in each of the EBPs, number of therapists per LOCI leader, the total number of inhabitants for each randomization unit, number of municipalities or districts for each unit, and number of inhabitants within the health trust served by the participating clinics. Power calculation based on 48 clinics showed that a difference at a little below .4 standard deviations would be detected with 80% power. The research group conducted the random allocation and enrollment, and assignment of participants.

Four clinics dropped out from the project during the initial phase of LOCI (one from cohort 1, two from cohort 2, and one from cohort 3) and were excluded from the primary analysis. Linear mixed-effects analysis with clustering on clinics demonstrated no significant differences in scores (*p* ≥ 0.564) between therapists in dropout and participating clinics regarding baseline scores on implementation leadership, transformational leadership, or implementation climate. Two clinics merged. The final sample consisted of 47 first-level leaders from 43 different child and adult clinics (there are more leaders than clinics due to a change in leadership in three clinics). Cohort 1 consisted of 14 clinics (16 leaders and 314 therapists), cohort 2 of 14 clinics (14 leaders and 231 therapists), and cohort 3 of 15 clinics (17 leaders and 245 therapists). Please see participant flow in the CONSORT diagram as Supplementary Material.

The LOCI training sessions (2 days at baseline and 1 day at 4, 8, and 12 months) were carried out face-to-face at the Norwegian Center for Violence and Traumatic Stress Studies (NKVTS). During these trainings, first-level leaders were introduced to general and strategic leadership principles and implementation climate. The leaders received feedback reports based on 360° assessments on their leadership and their clinics’ implementation climate. Based on this, they developed individualized leadership development plans to improve leadership and climate, which were updated based on new feedback reports every fourth month. The first-level leader had weekly coaching calls by phone with a LOCI trainer to strategize actions to achieve the goals defined in the leadership development plan. Once a month, the individual coaching calls were replaced with group coaching calls within each cohort.

The first organizational strategy meeting, which involved first-level leaders and executive management, was conducted at each clinic following the first LOCI training sessions, whereas the following organizational strategy meetings (at month 4, 8, and 12) were conducted through digital platforms. Consistent with the LOCI strategy focus on alignment of first-level leader activities and organizational supports, aggregated data on implementation climate and attitudes to EBPs were shared with executives and the LOCI leaders in each of the health trusts at every organizational strategy meeting. Based on the aggregated data, climate development plans to support the implementation and the first-level leaders in their implementation efforts on an organizational and executive management level were co-created and revised with health trust executives through 30 min monthly online meetings . The LOCI strategy was administrated separately in the child and adult clinics by two teams at the NKVTS. The team for adult clinics consisted of two clinical psychologists and one Ph.D., and the team for the child clinics included three clinical psychologists, one MA, and one Ph.D. Two of the five LOCI trainers in the child clinics were also responsible for training therapists in TF-CBT. Both teams were trained to deliver LOCI by the original LOCI developers. There were regular meetings between the Norwegian and US teams to discuss and review adaptations (such as context and design issues), translation of materials and measures, and fidelity to the LOCI protocol. LOCI’s developer (GAA) participated in the first LOCI workshops and follow-up workshops with both teams and attended and provided feedback on meetings with health trust executives. In addition, the Norwegian LOCI trainers had regular meetings to discuss the progress during the project period.

We collected data from all participating clinics throughout the study period, consistent with the stepped-wedge design. Data were collected using the Norwegian Centre for Research Data (NSD WebSurvey). There were six total data collection points (baseline in July 2018 and every 4 months until April 2020). The first two cohorts entered a sustainability phase at measurement times 5 and 6, respectively.

### Measures

The employees completed questionnaires about their perception of their leader and implementation climate for their clinic. For all scales, questions were tailored to evidence-based screening and treatment of PTSD, referring to the screening instruments and treatment methods being implemented.

### The implementation leadership scale (ILS)

ILS is a 12-item questionnaire measuring leadership for EBP implementation [21]. It consists of four subscales: (1) proactive leadership, (2) knowledgeable leadership, (3) supportive leadership, and (4) perseverant leadership. It is scored from 0 (not at all) to 4 (to a very great extent). The total ILS score was created by computing the mean of the four subscales. Individuals who had data on half or more of the items in each subscale were included. The scale demonstrated excellent psychometric properties (12-items; α = 0.955, CI (95%) = 0.945 – 0.963). The ILS is freely available at www.implementationleadership.com, and was translated by an independent research group at the Regional Center for Children and Adolescent Mental Health (RBUP) in close collaboration with the developers of the scale. The ILS demonstrated good psychometric properties in the current study [41].

### The multifactor leadership questionnaire (MLQ)

MLQ is a 36-item questionnaire that is built on the full-range leadership theory [42]. It measures three leadership behaviors, including transformational, transactional, and non-leadership. Of these, the primary focus for this study was transformational leadership. Transformational leadership consists of four subscales (idealized influence, 8 items; inspirational motivation, 4 items; intellectual stimulation, 4 items; and individual consideration, 4 items). The other scales on the MLQ were also included in the analyses for comparison purposes. Transactional leadership (contingent reward, 4 items; active management-by-exception, 4 items; passive management-by-exception, 4 items) and non-leadership (laissez-faire, 4 items) consists of three and one subscales, respectively [43]. It is scored from 0 (not at all) to 4 (frequently, if not always). While the transformational leadership scale is psychometrically supported in the literature [44], the other scales covary differently both theoretically and empirically from standard psychometric representations [45, 46]. We therefore created a total score for transformational leadership by calculating the mean scores across the four subscales while analyzing the other subscales of transactional and non-leadership separately. Participants with data on two or more of the items in each subscale were included.

Psychometric properties for transformational leadership were excellent (20-items; α = 0.958, CI (95%, bootstrapping based on 1000 samples) = 0.948 – 0.965) while the subscales for Transactional Leadership all had good item reliability, specifically contingent reward (4-items; α = 0.846, CI (95%, bootstrapping based on 1000 samples) = 0.813 – 0.871), active management-by-exception (4-items; α = 0.881, CI (95%, bootstrapping based on 1000 samples) = 0.859 – 0.898), passive management-by-exception (4-items; α = 0.842, CI (95%, bootstrapping based on 1000 samples) = 0.812 – 0.868), and laissez-faire leadership (4-items; α = 0.867, CI (95%) = 0.838 – 0.892). A license was obtained to use the MLQ. A Norwegian version was used in the current study [47]. The MLQ demonstrated good psychometric properties in the current study [41].

### The implementation climate scale (ICS)

The ICS is an 18-item questionnaire measuring a climate that supports EBP adoption and use in organizations [27]. It includes six subscales: (1) focus on EBP, (2) educational support for EBP, (3) recognition for EBP, (4) rewards for EBP, (5) selection for EBP, and (6) selection for openness. It is scored from 0 (not at all) to 4 (to a very great extent). Participants with data on two or more items in each subscale were included, and the total ICS score was calculated by computing a mean score of all subscales. The ICS showed very good psychometric properties (18-items; α = 0.894, CI (95%) = 0.873 – 0.910). The ICS is freely available at www.implementationleadership.com. A translated version into Norwegain was used [48]. ICS, with the exception of the Reward subscale, demonstrated good psychometric properties in the current study [48].

### The implementation climate measure (ICM)

To include a more global understanding of implementation climate, the ICM, a 6-item questionnaire measuring the general implementation climate in the organization, was also included [49]. It includes three subscales measuring what is (1) expected, (2) supported, and (3) rewarded when implementing a new practice. The scale is scored from 0 (not at all) to 4 (often, if not always). As each subscale only contains two items, participants had to have data on all items to be included in the analyses. The ICM total scale score was calculated by the mean scores of all subscales. It showed excellent psychometric properties (6-items; α = 0.918, CI (95%) = 0.901 – 0.932). We received permission to translate and use the ICM by the developers of the scale.

### Analyses

All data were exported from NSD WebSurvey to SPSS. The analyses were performed in R [50], using the nmle package [51] for the repeated measures. To assess the internal validity of ILS, MLQ, ICS, and ICM, Cronbach’s alpha was calculated using the cronbach.alpha function in the ltm package, including a 95% confidence interval using bootstrapping with 1000 samples to all stated confidence intervals for Cronbach’s alpha.

All analyses included data provided by therapists on their perception of general leadership, implementation leadership, and implementation climate. Data provided by leaders were excluded in the current study. In a repeated measures design, responses at the individual level (i.e., therapists) and responses from individuals within the same clinic are likely to be correlated. To account for the dependency in the data, we used linear mixed-effects models, which allows for irregularly spaced measurement time periods [52], and missing data within measurements [53], with fixed effects representing different linear changes before and during the LOCI intervention, and random effects for differences between clinics, and differences in level and slope between therapists. The random structure was simplified when necessary for model stability, as recommended [54]. The proportion of missing data was 3.3% for the ILS, 3.2% for the ICS, 2.7% for the ICM, 1.3 for the MLQ transformational leadership, 1.5% for contigent reward and laissez-faire, and 1.6% for management by exception – active and passive. Missing data was excluded from the models. The gap between the two linear fits, evaluated when LOCI began, represents the initial impact of LOCI training, where a positive value indicates improvement. Standardized versions of the initial impact, termed d, are computed by dividing by the square root of the combined variances for random effects in levels. If the post-LOCI slope is higher than the pre-LOCI slope, it indicates that the effects of LOCI training increase over time.

To examine possible differences between cohorts, training (i.e., received training in screening tools only or both screening tools and the EBPs for PTSD), and outpatient clinics (i.e., child or adult psychiatric care), we added categorical variables in the model for these in supplementary analyses. Separate parameters were included to test whether the LOCI training and the pre-and post- trajectories differed across child and adult clinics. Details on these supplementary analyses are only provided when there is a significant interaction.

## Results

**Fig. 1:**
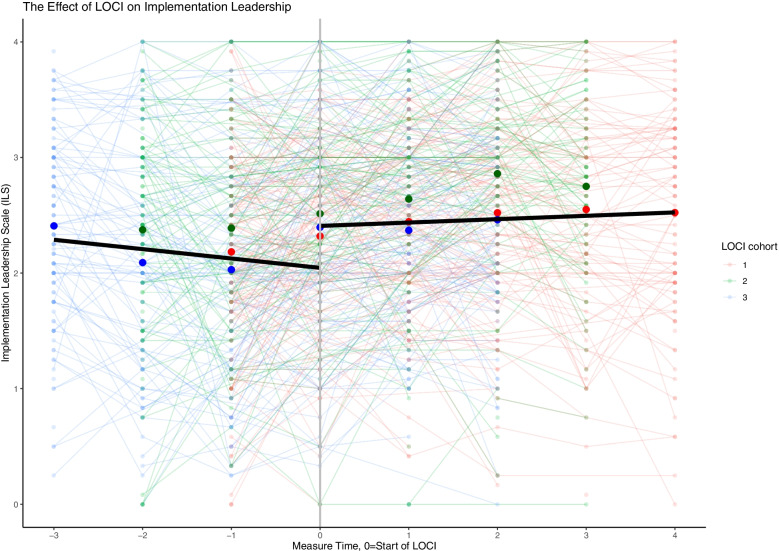
Staff-rated implementation leadership before and after the introduction of LOCI. Measure times − 3 to − 1 are the non-LOCI periods, while measure times 0 to 4 are the LOCI periods. Cohort 1 includes measure times − 1 to 4, cohort 2 includes measure times − 2 to 3, cohort 3 includes measure time − 3 to 2. The black line represents the estimated slope in the non-LOCI and LOCI periods. The large dots show the trajectories for each cohort (respectively) over time

**Fig. 2:**
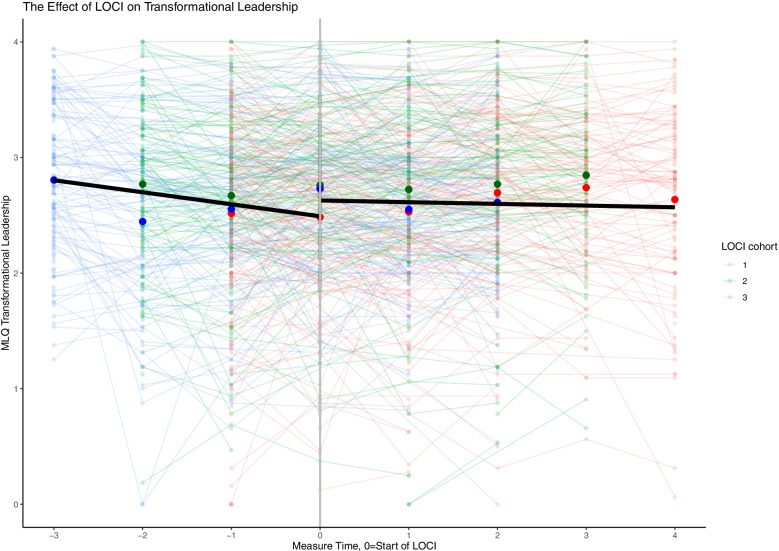
Staff-rated transformational leadership before and after the introduction of LOCI. Measure times − 3 to − 1 are the non-LOCI periods, while measure times 0 to 4 are the LOCI periods. Cohort 1 includes measure times − 1 to 4, cohort 2 includes measure times − 2 to 3, cohort 3 includes measure time − 3 to 2. The black line represents the estimated slope in the non-LOCI and LOCI periods. The large dots show the trajectories for each cohort (respectively) over time

**Fig. 3:**
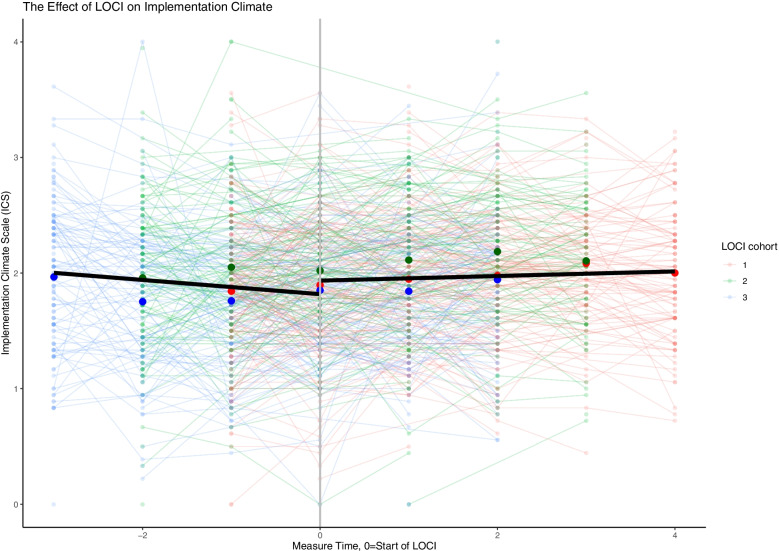
Staff-rated implementation climate (ICS) before and after LOCI. Measure times − 3 to − 1 are the non-LOCI periods, while measure times 0 to 4 are the LOCI periods. Cohort 1 includes measure times − 1 to 4, cohort 2 includes measure times − 2 to 3, cohort 3 includes measure time − 3 to 2. The black line represents the estimated slope in the non-LOCI and LOCI periods. The large dots show the trajectories for each cohort (respectively) over time

**Fig. 4:**
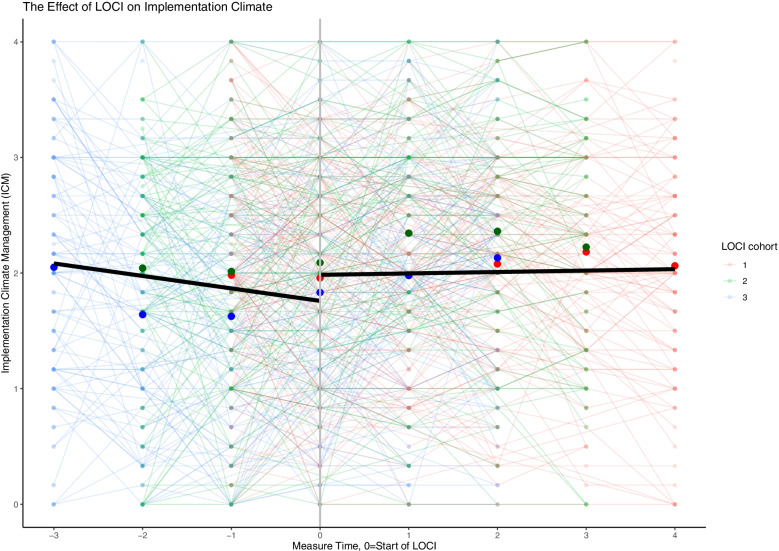
Staff-rated implementation climate (ICM) before and after LOCI. Measure times − 3 to − 1 are the non-LOCI periods, while measure times 0 to 4 are the LOCI periods. Cohort 1 includes measure times − 1 to 4, cohort 2 includes measure times − 2 to 3, cohort 3 includes measure time − 3 to 2. The black line represents the estimated slope in the non-LOCI and LOCI periods. The large dots show the trajectories for each cohort (respectively) over time

The findings across the three leadership measures are consistent. Across the three cohorts and prior to LOCI enrollment, there was a steady decrease in the therapist’s perception of implementation leadership, transformational leadership, and a supportive implementation climate. When LOCI was introduced, there was a significant increase in leadership and climate scores over time across all outcomes (Table 3; Figs. 1, 2, 3 and 4). These findings are described in more detail below.

**Table 3:** The effect of LOCI based on mixed effects analyses

Effect	Estimate	95% CI		***P***
		*LL*	*UL*	
**Implementation Leadership Scale (ILS)**				
Value when LOCI starts				
Non-LOCI	2.05	1.89	2.21	< 0.001
LOCI	2.41	2.27	2.54	< 0.001
Difference LOCI-non-LOCI	0.36	0.25	0.47	< 0.001
Slope				
Non-LOCI	−0.121	−0.182	− 0.060	< 0.001
LOCI	0.029	0.000	0.058	0.044
Difference LOCI- non-LOCI	0.151	0.084	0.217	< 0.001
**Transformational Leadership (MLQ)**				
Value when LOCI starts				
Non-LOCI	2.49	2.36	2.62	< 0.001
LOCI	2.63	2.52	2.74	< 0.001
Difference LOCI-control	0.14	0.05	0.22	0.001
Slope				
Non-LOCI	−0.104	−0.150	−0.057	< 0.001
LOCI	−0.015	− 0.373	0.008	0.202
Difference LOCI- non-LOCI	0.089	0.039	0.140	< 0.001
**Implementation Climate Scale (ICS)**				
Value when LOCI starts				
Non-LOCI	1.82	1.72	1.91	< 0.001
LOCI	1.93	1.86	2.01	< 0.001
Difference LOCI- non-LOCI	0.12	0.04	0.20	0.004
Slope				
Non-LOCI	−0.062	−0.107	−0.016	0.008
LOCI	0.020	−0.001	0.041	0.062
Difference LOCI- non-LOCI	0.081	0.032	0.131	0.001
**Implementation Climate Measure (ICM)**				
Value when LOCI starts				
Non-LOCI	1.76	1.62	1.90	< 0.001
LOCI	1.98	1.88	2.09	< 0.001
Difference LOCI-non-LOCI	0.23	0.10	0.35	< 0.001
Slope				
Non-LOCI	−0.108	−0.179	−0.040	0.002
LOCI	0.012	−0.021	0.046	0.470
Difference LOCI - non-LOCI	0.120	0.043	0.198	0.002

Table 3 provides the main results of the analyses for the study. For each outcome of interest, the key implementation results are the difference between LOCI and non-LOCI sites at the first measurement point after LOCI for each cohort (i.e., the immediate difference), followed by the slope for that outcome for all available data points for LOCI versus non-LOCI (i.e., change after initial implementation). The figures display considerable variation across therapists using thin lines for all therapist scores in a spaghetti plot. Each figure represents a single implementation outcome (i.e., implementation leadership in Fig. 1, transformational leadership in Fig. 2, and implementation climate in Fig. 3). For each outcome in Table 3, the estimates and comparison for the “value when LOCI starts” correspond to the intercept estimate at time 0 in the figures. The figures’ slope estimates and comparisons correspond to the dark black lines for the non-LOCI and LOCI periods.

### The effect of LOCI on implementation leadership (ILS)

As shown in Table 3 and Fig. 1, there was a positive initial effect of LOCI on implementation leadership relative to non-LOCI (difference = 0.36, *p* <  0.001, *d* = 0.42). This indicates that the therapists scored their leaders higher on implementation leadership following inclusion in LOCI. The slope for the pre-LOCI data was significant and negative, meaning that therapists rated implementation leadership steadily lowered their ratings over time before starting LOCI. Over time, the slope for the LOCI sites was significant and positive, meaning that therapists rated implementation leadership steadily increased their ratings after the initial increase at the start of training. These two slopes were significantly different from each other, further indicating that LOCI disrupted the early pattern of decreasing leadership ratings.

### The effect of LOCI on transformational leadership (MLQ)

There was a positive initial effect of LOCI on transformational leadership relative to the non-LOCI (diff = 0.14, p <  0.001, *d* = 0.19) (Table 3; Fig. 2). This suggests a significant increase in therapist’s rates on transformational leadership once the leaders initiated their participation in LOCI. The slope for the pre-LOCI data was significant and negative, meaning that therapists rated transformational leadership steadily lower over time before starting LOCI. The slope for the post-LOCI data was not significant, meaning that transformational leadership scores may have stayed consistent after the initial increase once LOCI was initiated. These two slopes were significantly different from each other. There was no significant change in therapists’ rates of transactional leadership (contingent reward (diff = 0.09, *p* = 0.113, *d = 0.10*, active (diff = − 0.03, *p* = 0.684, *d = − 0.03*) or passive management-by-exception (diff = − 0.04, *p* = 0.478, *d = − 0.05)* or laissez-faire leadership (diff = 0.03, *p* = 0.572, *d = 0.04*).

The three-way interaction between outpatient clinics, time for inclusion in LOCI, and measure time for adult and child outpatient clinics was significant for transformational leadership (*p* < .001). Examination of the coefficients for the effect of LOCI on transformational leadership shows that most of the effect was due to a change in the adult psychiatric clinics (Table 4). In particular, neither initial nor slope changes on transformational leadership were significant for child clinics, whereas both were significant and favored LOCI for the adult clinics.

**Table 4:** Mixed effects analysis on adult and child clinics (MLQ transformational leadership) and training in screening only compared to training in screening and EBP for PTSD (ICS)

Effect	Estimate	95% CI		*P*
		*LL*	*UL*	
**Adult and child outpatient clinics – MLQ Transformational Leadership**				
Child outpatient clinics				
Value when LOCI starts				
Non-LOCI	2,67	2.47	2.86	< 0.001
LOCI	2.66	2.50	2.82	< 0.001
Difference LOCI- non-LOCI	−0.01	−0.14	0.12	0.928
Slope				
Non-LOCI	0.001	−0.075	0.076	0.985
LOCI	−0.029	−0.006	0.004	0.089
Difference LOCI- non-LOCI	−0.029	− 0.566	−0.012	0.479
Adult outpatient clinics				
Value when LOCI starts				
Non-LOCI	2.38	2.19	2.56	< 0.001
LOCI	2.60	2.44	2.76	< 0.001
Difference LOCI-non-LOCI	0.22	0.12	0.33	< 0.001
Slope				
Non-LOCI	−0.168	−0.227	−0.109	< 0.001
LOCI	−0.002	−0.033	0.029	0.903
Difference LOCI- non-LOCI	0.166	0.100	0.232	< 0.001
**Training in screening only versus screening and treatment methods – ICS**				
Training in evidence-based screening only				
Value when LOCI starts				
Non-LOCI	1.91	1.79	2.03	< 0.001
LOCI	1.94	1.86	2.02	< 0.001
Difference LOCI- non-LOCI	0.027	−0.08	0.13	0.630
Slope				
Non-LOCI	−0.001	− 0.063	0.061	0.970
LOCI	0.017	−0.012	0.046	0.244
Difference LOCI- non-LOCI	0.018	−0.049	0.086	0.595
Training in evidence-based screening and treatment methods				
Value when LOCI starts				
Non-LOCI	1.71	1.57	1.85	< 0,001
LOCI	1,94	1,84	2,03	< 0,001
Difference LOCI- non-LOCI	0.23	0.11	0.35	< 0,001
Slope				
Non-LOCI	−0.132	−0.198	−0.065	< 0.001
LOCI	0.023	−0.008	0.053	0.143
Difference LOCI- non-LOCI	0.154	0.082	0.226	< 0.001

### The effect of LOCI on implementation climate

There was an initial, significant positive effect of LOCI on implementation climate as measured by the Implementation Climate Scale (ICS) relative to the non-LOCI (diff = 0.12, *p* <  0.001, *d* = 0.19), with practitioners reporting higher scores on implementation climate after the introduction of LOCI (Table 2). The pre-LOCI slope was significant and negative, meaning that ICS scores decreased over time before starting LOCI. The slope for the post-LOCI data was not significant, meaning that ICS scores may have stayed relatively consistent after the initial increase once LOCI was initiated. These two slopes were significantly different from each other.

The results for the Implementation Climate Measure (ICM) were similar to the ICS results. There was an initial, significant positive effect of LOCI on implementation climate as measured by the ICM relative to the non-LOCI (diff = 0.23, *p* = 0.004, *d* = 0.24), which indicates that participants reported higher ICM scores after the introduction of LOCI (Table 2; Fig. 3). The slope for the pre-LOCI data was significant and negative, meaning that ICM scores decreased over time before starting LOCI, and the slope for the post-LOCI data was not significant. These two slopes were significantly different from each other.

### Training in screening only versus training in screening and EBP for PTSD

**Fig. 5:**
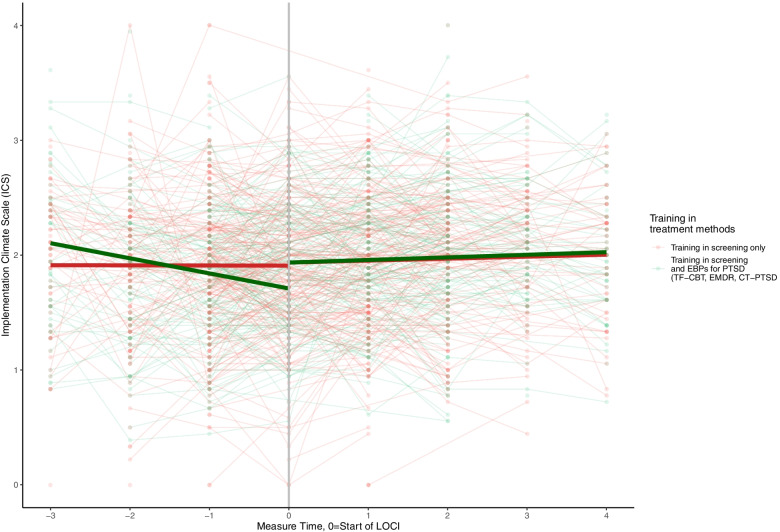
Staff-rated implementation climate among providers receiving training in screening only versus training in screening and EBP for PTSD treatment. Measure times − 3 to − 1 are the non-LOCI periods, while measure times 0 to 4 are the LOCI periods. Cohort 1 includes measure times − 1 to 4, cohort 2 includes measure times − 2 to 3, cohort 3 includes measure time − 3 to 2. The black line represents the estimated slope in the non-LOCI and LOCI periods. The large dots show the trajectories for each cohort (respectively) over time

There were no significant differences between individuals who had received training in screening only versus those who received training in screening and the EBPs for PTSD (TF-CBT, EMDR, CT-PTSD) on ILS, MLQ, or ICM (Table 3). However, there were significant differences between the two groups for ICS, suggesting that those who had received training in both screening and EBP for PTSD may have largely contributed to the effect of LOCI on the ICS (*p* = 0.007) (Table 4; Fig. 5).

## Discussion

The translation of research into practice remains a challenge within mental health systems. Despite the documented importance of leaders in this respect, there is a lack of knowledge on the effectiveness of leadership development programs related to the implementation of EBPs. Mental health care service practitioners experience high job demands and challenges such as burnout and turnover [7]. To successfully implement and sustain EBPs, clinic level and broader organizational leadership support are essential [25]. This study provides new knowledge on how to improve staff-rated leadership and climate to enhance effective implementation of evidence-based practices. Building on past research on LOCI in US contexts, this study expands the implementation research field by demonstrating the utility of this implementation strategy in a Norwegian mental health setting. Therapists’ experiences of leadership and implementation climate decreased over time following training of therapists in evidence-based practices while significantly increasing when LOCI was introduced. This increase was sustained throughout the project period, compared to non-LOCI conditions which demonstrated a steady decrease in scores before LOCI was introduced. This suggests that clear implementation strategies are vital to achieving good leadership and a positive implementation climate.

The current study demonstrated that following training in EBPs, the therapists’ perception of implementation leadership and implementation climate decreased steadily over time. However, when the leaders received the LOCI intervention, the therapists’ reports of implementation leadership and climate significantly increased. Thus, in line with our hypotheses, LOCI facilitated implementation leadership and a positive implementation climate. These findings also indicate that training therapists in EBPs without having a clear implementation strategy and leadership support might have a detrimental effect and that types of implementation support provided by LOCI are necessary. If this is a generalizable finding, it should impact how we implement EBPs and serve as a strong argument against dissemination through therapist-trainings only. The timing of when to introduce a strategy such as LOCI should also be considered. If introduced in the preparation phase of an implementation process, and before the training of therapists in EBPs, the leaders can be more prepared to facilitate the implementation process from the beginning. It is possible that the scores would have been higher if LOCI had been introduced earlier in the preparation phase (before the active implementation phase) – an empirical question which we encourage future studies to investigate. Moreover, for the LOCI intervention to have long-term implications for mental health systems, the gains in leadership and climate must be maintained over time. These results indicate that the effects were maintained for the length of the project period (24 months).

There was a significant positive effect of LOCI on therapists’ reports of transformational leadership. However, subsequent analysis showed that the adult clinics might have mainly accounted for this effect. There was a larger drop in therapist-rated scores on transformational leadership in the adult clinics prior to engagement in LOCI, which means that there was more room for change. Also, therapists at the child clinics received approximately 40 cases of coaching as part of their TF-CBT training, whereas the adult therapists received 10 h of coaching as part of their EMDR and CT-PTSD training. It might be that the different training models in the adult versus the child clinics accounted for the different patterns in the data before the introduction of LOCI at baseline, as well as the different trajectories throughout the project period. For example, the therapists in the child clinics who received more coaching might have felt more connected with the other therapists [55], which could have affected their need for, and perception of, leadership support. Another explanation could be that the LOCI trainers in the adult clinics focused more on developing general leadership skills among the first-level leaders, whereas the LOCI trainers in the child clinics focused more on the implementation of the PTSD treatment method as some of them also were the TF-CBT trainers.

Laissez-faire leadership or passive-avoidant leadership may have detrimental effects on implementation efforts [5], and as such one might expect laissez-faire leadership to be reduced when active leadership increases. Yet, there were no significant effects of LOCI on the non-leadership (laissez-faire leadership) or transactional leadership dimensions. This might be due to LOCI emphasizing improvements in first-level leaders’ transformational leadership as an additive to transactional leadership behaviors, and secondly, because the leaders generally scored low on non-leadership.

The group receiving training in both screening and EBPs for PTSD contributed to the effect of LOCI on implementation climate as measured by the ICS. The questionnaire targeted screening and EBPs for PTSD, and it might be that the therapists who were trained in screening only perceived that the questions were not as relevant as for the practitioners that were also trained in the EBPs for PTSD. The results might also signal that those trained in both screening and EBPs for PTSD exhibit a larger need for implementation climate support. The scores on implementation climate were quite high at baseline among those trained in both screening and EBPs for PTSD, which might indicate enthusiasm over the new project. When therapists perceive the implementation climate as good, it signals that EBP is a lasting prioritization within the organization [26]. Following the initial enthusiasm, it might be that those trained in both screening and EBPs for PTSD treatment experienced a drop in implementation climate over time until the LOCI intervention was introduced. On the other hand, the baseline scores were quite low among the therapists trained in screening only. This might have been an expression of a wait-and-see attitude at the start of the project, which was strengthened when they experienced that the implementation was more than a passing trend.

There was a significant interaction effect for implementation climate as measured by the ICS, but not the ICM, suggesting that mainly those who had received training in both screening and EBP for PTSD contributed to the effect of LOCI on the ICS. The ICS subscales are more specific regarding implementation climate dimensions (focus on EBP, educational support, recognition, rewards, selection for EBP, and openness for EBP). ICM is a more global measure of implementation climate. Hence, it may be that LOCI had a positive effect on general implementation climate, whereas only those trained in the EBPs experience a more positive implementation climate for specific dimensions.

This is the first study to investigate the effect of LOCI in a health care setting outside of the USA. It is a strength that the study involved clinics all over Norway that implemented screening and EBP treatment. The findings might be generalizable to similar settings. The implementation context was characterized by supportive policies, governmental funding and high level of trust in the population generally. More knowledge is needed on the adaptation and utilization of LOCI in decentralized and resource-constrained contexts. While the use of multiple assessment times strengthens methodological rigor, potentially increased respondent burden as a result may have impacted their responses. The next step would be to examine whether and how transformational leadership, implementation leadership and implementation climate work as implementation mechanisms between other implementation, service, and patient outcomes. Future studies should investigate whether LOCI, through improved implementation leadership and climate, contributes to increased use of EBPs—and ultimately improved client outcomes. A parallel randomized controlled trial design might be considered to mitigate the possible limitations of a stepped-wedge design, e.g. related to model misspecification [56].

## Conclusions

This study contributed novel knowledge about the effect of the LOCI intervention on key factors highlighted as important for successful implementation of EBPs – namely, leadership and climate. Implementation and transformational leadership and implementation climate were more positively evaluated after the leaders were introduced to the LOCI intervention, and this was sustained throughout the project period, whereas non-LOCI conditions demonstrated a steady decrease in therapist-rated scores before LOCI was introduced. LOCI seems like an appropriate implementation strategy for first-level leaders to achieve better EBP implementation and sustainment within mental health care services.

## Supplementary Information

**Additional file 1.** CONSORT 2010 Flow Diagram.

## Data Availability

The datasets will be available from the corresponding author on reasonable request.

## Abbreviations

LOCI: Leadership and Organizational Change for Implementation
EBP: Evidence-based practices
PTSD: Posttraumatic stress disorders
EMDR: Eye Movement and Desensitization Reprocessing
CT-PTSD: Cognitive Therapy for PTSD
TF-CBT: Trauma-Focused Cognitive Behavioral Therapy
MLQ: The Multifactor Leadership Questionnaire
ILS: Implementation leadership scale
ICS/ICM: Implementation climate scale /implementation climate measure
