# Supplementary material for: A stepped-wedge randomized trial investigating the effect of the Leadership and Organizational Change for Implementation (LOCI) intervention on implementation and transformational leadership, and implementation climate
Source: BMC Health Serv Res. 2022 Mar 4;22:298. doi: 10.1186/s12913-022-07539-9 (PMC8895588; doi:10.1186/s12913-022-07539-9)
Supplement: Supplementary file 1 — Additional file 1. CONSORT 2010 Flow Diagram. [file 12913_2022_7539_MOESM1_ESM.doc]

**
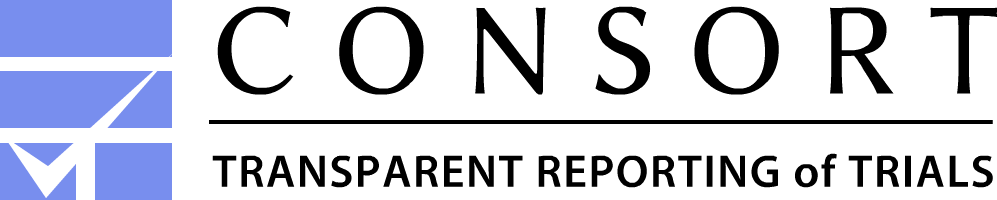
**

**CONSORT 2010 Flow Diagram**

**Allocation**

**Analysis**

**Enrollment**

Recruited clinics (n=48) and leaders (n=48)

Allocated to cohort 1 (n=16 clinics, 16 leaders)

♦ Received allocated intervention (n=14* clinics, 16 leaders)

♦ Did not receive allocated intervention (drop-out) (n=1 clinic, 1 leader)

Allocated to cohort 3 (n=16 clinics, 16 leaders)

♦ Received allocated intervention (n=15 clinics, 17 leaders)

♦ Did not receive allocated intervention (drop-out) (n=1 clinic, 1 leader)

Randomized (n=48 leaders)

Allocated to cohort 2 (n=16 clinics, 16 leaders)

♦ Received allocated intervention (n=14 clinics, 14 leaders)

♦ Did not receive allocated intervention (drop-out) (n=2 clinics, 2 leaders)

Analysed (n=14 clinics, 14 leaders)

Analysed (n=14 clinics, 16 leaders)

Analysed (n=15 clinics, 17 leaders)
